# Risk factors associated with human echinococcosis: a systematic review and meta-analysis

**DOI:** 10.3389/fvets.2024.1480579

**Published:** 2024-11-25

**Authors:** Fahmi H. Kakamad, Khanda A. Anwar, Harem K. Ahmed, Imad J. Habibullah, Hemn H. Kaka Ali, Hawkar A. Nasralla, Hiwa O. Abdullah, Soran H. Tahir, Honar O. Kareem, Ali H. Hasan, Dana T. Gharib, Hoshmand R. Asaad, Ayoob A. Mohammed, Berun A. Abdalla, Deari A. Esmaeil, Rezheen J. Rashid, Karokh F. Hamahussein

**Affiliations:** ^1^Scientific Affairs Department, Smart Health Tower, Madam Mitterrand Street, Sulaymaniyah, Iraq; ^2^College of Medicine, University of Sulaimani, Madam Mitterrand Street, Sulaymaniyah, Iraq; ^3^Kscien Organization for Scientific Research (Middle East Office), Sulaymaniyah, Iraq; ^4^Gastroenterology and Hepatology Teaching Hospital, Sulaymaniyah, Iraq; ^5^Department of Radiology, Hiwa Cancer Hospital, Sulaymaniyah, Iraq

**Keywords:** alveolar echinococcosis, cystic echinococcosis, risk factors, *Echinococcus granulosus*, hydatid cyst, zoonosis

## Abstract

**Introduction:**

Echinococcosis is a widespread zoonotic disease caused by tapeworms of the Echinococcus genus, manifesting in mature or larval forms. Cystic echinococcosis (CE) and alveolar echinococcosis (AE) are the primary types affecting humans, linked, respectively, to *Echinococcus granulosus* and *Echinococcus multilocularis*. This study is a systematic review and meta-analysis of the risk factors associated with CE and AE in humans.

**Methods:**

Relevant English publications were found through a thorough search of eligible databases. The inclusion criteria focused on cross-sectional and case–control studies investigating risk factors for human echinococcosis. Collected data included author, country, study design, demographics, sample size, literacy, occupation, drinking water source, dog ownership, and hand hygiene.

**Results:**

A total of 1,594 studies were found in the initial search, with only 36 papers (involving 1,207,436 cases) meeting the inclusion criteria. Most of the study population (99.35%) showed no echinococcosis infection, while 0.65% were infected. Of the infected cases, 77.92% had CE, while 22.08% had AE. Among 629,996 (52.18%) females, 4,830 (0.76%) were infected, compared to 2,968 (0.52%) infections among 565,872 (46.86%) males (*p* < 0.001). Rural areas, low education levels, agricultural/livestock workers, dog owners, water sources, and poor hand hygiene were all significantly associated with the infection (*p* < 0.05).

**Conclusion:**

Echinococcosis remains a global health concern, particularly among rural residents, those with lower education, agricultural workers, and dog owners. Targeted public health measures, including improved hygiene practices and access to clean water, are essential to reducing its impact.

## 1 Introduction

Echinococcosis is a widespread zoonotic illness caused by the mature or larval forms of tapeworms found in the *Echinococcus genus* (Taeniidae family). This disease is among the most ancient ones recorded, origin tracing back to the time of Hippocrates (1). The larval infection, known as hydatid disease or hydatidosis, is identified by the gradual development of metacestode (hydatid) cysts within the intermediate host over an extended period (2). The disease occurs worldwide, with a notable prevalence in regions like Eastern Europe, South Africa, the Middle East, South America, Australia, and the Mediterranean, where cattle and sheep farming is common (1). Cystic echinococcosis (CE) and alveolar echinococcosis (AE) represent the predominant types of human disease. They are triggered by the tapeworms *Echinococcus granulosus* and *Echinococcus multilocularis*, respectively (3). Typically, CE persists within the domestic cycle involving dogs and domestic ungulates. It remains a prevalent zoonosis in rural regions where humans adopt domesticated dogs. On the other hand, AE is primarily sustained through a wild cycle involving foxes and rodents, which may have connections with domestic dogs and cats (4). Human infections typically occur incidentally through ingesting the parasite eggs from infected definitive hosts or exposure to other sources like consuming contaminated food or water, direct physical contact, or interacting with infected animals like dogs (5, 6). This disease commonly affects the liver (70%) and lungs (20%); however, there are also instances of the disease occurring in less typical locations, such as the neck, kidneys, heart, vascular system, bladder, and thyroid gland (7–9). This study is a systematic review and meta-analysis of the risk factors associated with CE and AE in humans.

## 2 Method

### 2.1 Study design

This meta-analysis followed the Preferred Reporting Items for Meta-Analyses (PRISMA) guidelines.

### 2.2 Data sources and search strategy

A thorough search in “Google Scholar” and “PubMed” was conducted to identify all relevant publications in English. The search combined terms using Boolean operators, and the keywords were echinococcosis OR echinococci OR hydatidosis OR echinococcus AND transmission OR factor OR prevalence.

### 2.3 Eligibility criteria

The inclusion criteria encompassed cross-sectional and case–control studies concentrating on risk factors linked to human echinococcosis. Studies were excluded if they: (1) were non-English; (2) only had an abstract available; (3) were case reports or case series; (4) were non-articles, review articles, editorials, or opinion pieces lacking primary data; and (5) lacked a control group. All the included studies were assessed for eligibility (10).

### 2.4 Study selection and data extraction

The titles and abstracts of the identified studies underwent initial screening before a thorough full-text assessment for eligibility. The data gathered from the studies included the first author’s name, country, type of study design, gender, residency, sample size, number of infected and healthy cases, level of education, occupation, drinking water source, dog ownership, and hand hygiene.

### 2.5 Statistical analysis

Data collection and organization were conducted using Microsoft Excel (2019), while data analysis was performed using Statistical Package for Social Sciences (SPSS) software (v.25). The data were presented in frequency, and percentage. The qualitative analysis was performed utilizing the Chi-squared test, with a significant level determined at a *p*-value of 0.05 or less.

## 3 Results

**Figure 1:**
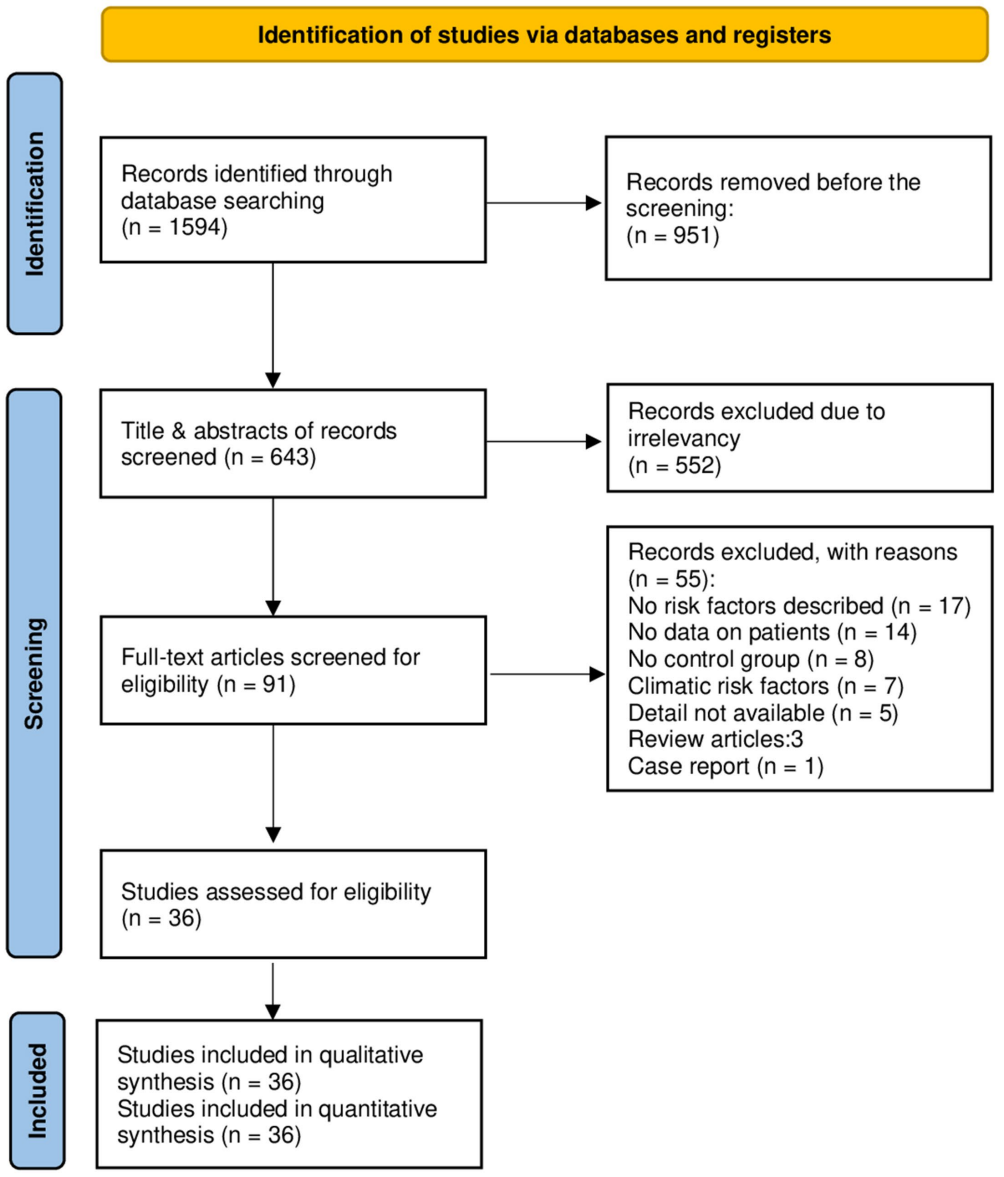
The PRISMA flow chart detailing the process of study selection.

A total of 1,594 studies were found in the initial search, 951 of which were directly removed due to irrelevancy. Then, the titles and abstracts of the remaining 643 papers were screened, of which 552 papers were excluded. The full text of the remaining 91 papers was read, of which 55 papers were excluded, and then finally, 36 papers (3, 6, 11–44) involving 1,207,436 cases were compatible with the inclusion criteria (Figure 1). Among these included studies, 27 (75%) were cross-sectional, while 9 (25%) were case–control. The vast majority of cases (99.35%) were non-infected, while 7,815 cases (0.65%) were identified as infected. Specifically, 6,089 cases (77.92%) presented with CE, while only 1,726 cases (22.08%) had AE (Table 1). China reported the largest number of echinococcosis cases, with 5,748 infections, representing 73.55% of the total cases recorded. Iran ranks next, with 803 cases, accounting for 10.27% (Table 2). The majority of cases, 6,262 (80.13%), were diagnosed using a combination of serology and ultrasound or other imaging methods. Serology alone was used in 917 cases (11.74%), ultrasound alone in 303 (3.87%), and in 333 cases (4.26%), the diagnostic method was unrecorded. In terms of gender distribution, the total number of females was 629,996 (52.18%), with 4,830 (0.77%) being infected, whereas the total number of males was 565,872 (46.86%), with 2,968 (0.52%) infected (*p* < 0.001). However, the gender of 11,568 individuals (0.96%) was not specified (Table 3). Furthermore, among the rural population totaling 25,369 individuals, 1,594 cases (6.29%) were infected, in contrast to 268 cases (2.70%) among the urban population of 9,893 individuals (*p* < 0.001). Out of participants with lower educational attainment, 5,392 (0.73%) were infected, whereas among those with higher educational levels, only 912 (0.22%) were infected (*p* < 0.001). In the occupational context, 5,640 cases (0.62%) of agricultural/livestock workers and 1,483 cases (0.57%) in other occupations were infected, with a statistically significant difference (*p* = 0.012). Moreover, 1,424 (6.24%) of dog owners were infected, while 682 (3.57%) of non-dog owners had the infection (*p* < 0.001). The infection rate among participants consuming public water supply was 272 cases (1.60%), whereas among those using alternative water sources, 94 cases (2.54%) were infected (*p* < 0.001). Participants adhering to proper hand hygiene practices before meals had a lower infection rate (1.14%) compared to those with inadequate hand hygiene (2.80%; *p* < 0.001; Table 4).

**Table 1:** The raw data of the included studies.

Author	Study design	Country	Total Cases	Infected	Non-infected	Gender					Resident				
						M+	M-	F+	F-	NA	Rural +	Rural -	Urban +	Urban -	NA
Acosta-Jamett et al. (11)	Cross-sectional	Chile	384	10	374	5	125	5	249	0	10	374	0	0	0
Acosta-Jamett et al. (12)	Cross-sectional	Chile	2,439	39	2,400	17	871	22	1,529	0	24	737	15	1,663	0
Ahmed et al. (13)	Cross-sectional	Sudan	305	20	285	7	105	13	180	0	NA	NA	NA	NA	305
Akalin et al. (14)	Cross-sectional	Turkey	1,133	78	1,055	29	501	49	554	0	78	1,055	0	0	0
Bai et al. (15)	Cross-sectional	China	882	99	783	33	NA	66	NA	783	99	783	0	0	0
Barati et al. (16)	Cross-sectional	Iran	1,232	12	1,220	4	520	8	700	0	NA	NA	NA	NA	1,232
Bingham et al. (17)	Cross-sectional	Argentina	340	33	307	11	66	22	241	0	NA	NA	NA	NA	340
Bitrus et al. (18)	Cross-sectional	Nigeria	187	6	181	3	108	3	73	0	NA	NA	NA	NA	187
Carmona et al. (19)	Cross-sectional	Florida & Uruguay	9,521	156	9,365	70	4,366	86	4,999	0	NA	NA	NA	NA	9,521
Craig et al. (20)	Cross-sectional	China	2,479	84	2,395	32	1,370	52	1,025	0	84	2,395	0	0	0
Dorjsuren et al. (21)	Cross-sectional	Mongolia	1829	87	1742	30	547	57	1,195	0	NA	NA	NA	NA	1829
Harandi et al. (22)	Cross-sectional	Iran	1,062	77	985	4	183	73	802	0	NA	NA	NA	NA	1,062
Kashinahanji et al. (23)	Cross-sectional	Iran	400	5	395	3	151	2	244	0	4	188	1	207	0
Khabisi et al. (24)	Cross-sectional	Iran	551	22	529	7	163	15	366	0	22	529	0	0	0
Ma et al. (3)	Cross-sectional	China	1,150,680	5,216	1,145,464	2099	545,192	3,117	600,272	0	NA	NA	NA	NA	1,150,680
Moshfe et al. (25)	Cross-sectional	Iran	1,005	81	924	11	216	70	708	0	81	924	0	0	0
Muhtarov (26)	Cross-sectional	Bulgaria	642	8	634	4	4	298	336	0	8	634	0	0	0
Ok et al. (27)	Cross-sectional	Turkey	6,093	9	6,084	NA	NA	NA	NA	6,093	5	1848	4	4,236	0
Othieno et al. (28)	Cross-sectional	Uganda	3,066	71	2,995	24	865	47	2,130	0	71	2,995	0	0	0
Rafiei et al. (29)	Cross-sectional	Iran	3,446	475	2,971	176	1,105	299	1866	0	475	2,971	0	0	0
Romig et al. (30)	Cross-sectional	Germany	2,540	60	2,480	31	1,161	29	1,319	0	60	2,480	0	0	0
Safarpour et al. (31)	Cross-sectional	Iran	1,500	131	1,369	52	697	79	672	0	NA	NA	NA	NA	1,500
Tamarozzi et al. (32)	Cross-sectional	Peru	1,304	41	1,263	13	365	28	898	0	NA	NA	NA	NA	1,304
Uchiumi et al. (6)	Cross-sectional	Argentina	892	42	850	24	354	18	496	0	18	192	24	658	0
Wang et al. (33)	Cross-sectional	China	3,676	66	3,610	NA	NA	NA	NA	3,676	NA	NA	NA	NA	3,676
Wang et al. (34)	Cross-sectional	China	705	60	645	25	325	35	320	0	60	645	0	0	0
Wang et al. (35)	Cross-sectional	China	7,138	223	6,915	91	3,334	132	3,581	0	164	4,614	59	2,301	0
Anuk and Çantay (36),	Case–control	Turkey	189	87	102	42	47	45	55	0	66	76	21	26	0
Campos-Bueno et al. (37)	Case–control	Spain	254	127	127	58	58	69	69	0	91	94	36	33	0
Dowling et al. (38)	Case–control	Jordan	176	44	132	18	54	26	78	0	0	0	0	0	176
Kreidl et al. (39)	Case–control	Austria	105	21	84	14	NA	7	NA	84	NA	NA	NA	NA	105
Laivacuma et al. (40)	Case–control	Latvia	92	46	46	14	14	32	32	0	15	2	31	44	0
Larrieu et al. (41)	Case–control	Argentina	90	24	66	NA	NA	NA	NA	90	19	34	5	32	0
Moro et al. (42)	Case–control	Peru	96	32	64	NA	NA	NA	NA	96	32	64	0	0	0
Piarroux et al. (43)	Case–control	France	746	180	566	NA	NA	NA	NA	697	108	141	72	425	0
Schmidberger, et al. (44)	Case–control	Germany	257	43	214	17	37	26	177	0	NA	NA	NA	NA	257

M+: Infected males, M-: Non-infected males, F+: infected Females, F-: non-infected Females, Rural+: Infected Rural residents, Rural-: Non-infected rural residents, N/A: Not Available Urban+: Infected urban residents, Urban-: Non-Infected urban residents, NA: Not available.

**Table 2:** The number of echinococcosis cases identified in the current study across various countries.

Country	No. of infected cases	Percentage (%)
China	5,748	73.55%
Iran	803	10.27%
France	180	2.30%
Turkey	174	2.23%
Uruguay	156	2.00%
Spain	127	1.63%
Germany	103	1.32%
Argentina	99	1.27%
Mongolia	87	1.11%
Peru	73	0.93%
Uganda	71	0.91%
Chile	49	0.63%
Latvia	46	0.59%
Jordan	44	0.56%
Austria	21	0.26%
Sudan	20	0.26%
Bulgaria	8	0.10%
Nigeria	6	0.08%

**Table 3:** Basic characteristics of the included studies.

Variables	Frequency/Percentage
Healthy	1,199,621 (99.35%)
Infected	7,815 (0.65%)
Gender	
Male	565,872 (46.86%)
Infected	2,968 (0.52%)
Non-infected	562,904 (99.48%)
Female	629,996 (52.18%)
Infected	4,830 (0.77%)
Non-infected	625,166 (99.23%)
NA	11,568 (0.96%)
Type of echinococcosis	
Alveolar echinococcosis	1,726 (22.08%)
Cystic echinococcosis	6,089 (77.92%)
Diagnostic methods	
Serology	917 (11.74%)
Ultrasound	303 (3.87%)
Serology + ultrasound or other diagnostic imaging	6,262 (80.13%)
NA	333 (4.26%)
Residency	
Rural	25,369 (2.10%)
Infected	1,594 (6.29%)
Non-infected	23,775 (93.71%)
Urban	9,893 (0.82%)
Infected	268 (2.70%)
Non-infected	9,625 (97.30%)
NA	1,172,174 (97.08%)
Literacy	
Low education level	742,535 (61.50%)
Infected	5,392 (0.73%)
Non-infected	737,143 (99.27)
High education level	421,252 (34.89%)
Infected	912 (0.22%)
Non-infected	420,340 (99.78)
NA	43,649 (3.61%)
Occupation	
Agricultural/livestock worker	918,773 (76.10%)
Infected	5,640 (0.62%)
Non-infected	913,133 (99.38%)
Non-agricultural/livestock worker	259,799 (21.51%)
Infected	1,483 (0.57%)
Non-infected	258,316 (99.43%)
NA	28,864 (2.39%)
Owning dogs	
Dog owners	22,807 (1.89%)
Infected	1,424 (6.24%)
Non-infected	21,383 (93.76%)
Non-dog owners	19,098 (1.58%)
Infected	682 (3.57%)
Non-infected	18,416 (96.42%)
NA	1,165,531 (96.53)
Water source	
Public water supply	17,005 (1.41%)
Infected	272 (1.59%)
Non-infected	16,733 (98.40%)
Other water sources	3,706 (0.31%)
Infected	94 (2.54%)
Non-infected	3,612 (97.46%)
NA	1,186,725 (98.28%)
Hand hygiene	
Handwashing before meals	6,564 (0.54%)
Infected	75 (1.14%)
Non-infected	6,489 (98.86%)
Not handwashing before meals	2,250 (0.19%)
Infected	63 (2.80%)
Non-infected	2,187 (97.20%)
NA	1,198,622 (99.27%)

NA: Not available.

**Table 4:** The comparison between patient demographics, dog ownership, drinking water sources, and hand hygiene practices with human echinococcosis infection.

Variables		Total			*p*-value
			Infected	Non-infected	
Resident	Rural	25,369	1,594 (6.29%)	23,775 (93.71%)	< 0.001
	Urban	9,893	268 (2.70%)	9,625 (97.30%)	
Gender	Male	565,872	2,968 (0.52%)	562,904 (99.48%)	< 0.001
	Female	629,996	4,830 (0.77%)	625,166 (99.23%)	
Literacy	Illiterate or primary school education	742,535	5,392 (0.73%)	737,143 (99.27)	< 0.001
	secondary and high school education	421,252	912 (0.22%)	420,340 (99.78)	
Occupation	Agricultural/livestock workers	918,773	5,640 (0.62%)	913,133 (99.38%)	0.012
	Non-agricultural/livestock workers	259,799	1,483 (0.57%)	258,316 (99.43%)	
Owning dogs	Dog owners	22,807	1,424 (6.24%)	21,383 (93.76%)	< 0.001
	Non-dog owners	19,098	682 (3.57%)	18,416 (96.42%)	
Water source	Public supply	17,005	272 (1.59%)	16,733 (98.40%)	< 0.001
	Other water sources	3,706	94 (2.54%)	3,612 (97.46%)	
Hand hygiene	Hand washing before meals	6,564	75 (1.14%)	6,489 (98.86%)	< 0.001
	Not handwashing before meals	2,250	63 (2.80%)	2,187 (97.20%)	

## 4 Discussion

Human echinococcosis, a prevalent parasitic infection, presents a considerable health and economic burden to society, yet it remains largely neglected as a disease (4). This disease typically thrives in nations with extensive livestock farming regions. However, it has become a pressing global health issue, largely attributed to increasing immigration rates and travel activities (45). The present study revealed that China had the highest number of echinococcosis cases, comprising 73.55% of all recorded cases. Iran followed with 10.27%. In contrast, European countries such as France, Turkey, and Germany had lower infection rates, each contributing less than 3%. Other countries, including Mongolia, Peru, and African nations like Uganda and Sudan, accounted for even smaller proportions. This distribution highlights significant regional differences in the incidence of echinococcosis.

The progression of the infection is gradual, with the initial phase of primary infection typically asymptomatic. Small cysts that do not cause significant disease may remain symptomless for extended periods, potentially leading to incidental detection. Parasite eggs can remain viable in various environments for several months to years. The precise duration of the incubation period for CE is uncertain but likely spans many months to years. Symptoms may arise if the cysts rupture or exert pressure due to their size (2, 12). Typically, AE manifests at a later stage than the cystic form. Cases of AE are marked by an initial asymptomatic incubation period lasting 5–15 years, followed by a chronic course. If left untreated or inadequately managed, AE can lead to high fatality rates (2).

Regarding transmission and risk factors, domestic dogs serve as the primary definitive host for both echinococcus species, posing the most significant risk of transmitting both CE and AE to humans (46). In a study by Ahmed et al., individuals in contact with dogs were at least twice as prone to acquiring an echinococcal infection (13). Dogs become infected by consuming offal from livestock that contains cysts. Afterward, they excrete the parasite eggs in their feces, contaminating the environment, including soil, water, and pastures. Livestock get the infection by ingesting these eggs while grazing. Human infection typically arises from consuming contaminated food or water (6). However, a review by Possenti and colleagues indicated that the primary means of transmitting human CE appears to be through the contamination of hands with eggs of *E. granulosus* excreted by dogs, either directly or indirectly (47). In line with these findings, the current study found that owning a dog significantly increases the likelihood of contracting echinococcosis, and there was also a correlation between inadequate hand hygiene and echinococcosis infection.

Otero-Abad et al. found that females are at a higher risk of echinococcosis than males. This heightened risk is attributed to their greater involvement in household chores, such as food preparation and pet care, which increases their exposure to infected dogs, soil, and vegetables (4). The current study also revealed that females exhibited a significantly higher risk of developing echinococcosis compared to males.

Schantz et al. indicated that individuals who own livestock are three times more likely to be diagnosed with this disease compared to those who do not own livestock (48). Additionally, pastoralism stands out as the occupation carrying the highest risk of contracting both types of echinococcosis. This is attributed to pastoralists’ proximity to livestock, dogs, and wildlife host species (46). The present study indicated that agricultural and livestock workers faced a greater risk of infection with echinococcosis than individuals in other occupations.

Regarding education, Ma et al. demonstrated that lower levels of education are associated with an increased risk of echinococcosis infection (3). In line with this finding, the present study found a significant association between lower education levels and a higher incidence of echinococcosis infection. This may be caused by the fact that educated individuals have higher knowledge about the risk of the disease and the factors that can protect against infection. Additionally, higher-educated individuals typically reside in urban areas and have a lower chance of contact with livestock or hosts.

The overall prevalence of human CE is significantly linked with rural residency, older age, and consumption of non-piped water (12). Othieno et al. indicated that unprotected open spring water sources have been identified as a risk factor in the occurrence of CE. These water sources are often shared with livestock and dogs. The frequency of sharing water increases notably during periods of water scarcity, such as the dry season and drought (28). In this study, the infection rate of echinococcosis was notably higher among participants who consumed water from sources other than public or tap water. Rural residents exhibited a significantly higher incidence of echinococcosis than urban residents.

Early detection of AE and CE can significantly enhance the effectiveness of managing and treating these conditions (49). The definitive diagnosis of AE and CE is typically achieved through physical imaging techniques, including radiology, ultrasonography, computed tomography (CT) scan, and magnetic resonance imaging (MRI) (50, 51). Serological tests, including enzyme-linked immunosorbent assay (ELISA), demonstrate high specificity for diagnosing hydatid disease; however, a positive result does not accurately indicate the cyst’s location within the body. In contrast, imaging modalities provide precise visualization, allowing for the detection of cysts in specific sites. This limitation highlights the necessity of combining serological testing with imaging to confirm both the diagnosis and the exact location of the hydatid cyst (52). In the present study, most cases (80.13%) were diagnosed through imaging techniques and serological testing together.

Case series and small clinical trials indicate a mortality rate of 2–4% for CE, but this rate significantly rises with inadequate treatment and care. Human AE is estimated to cause approximately 18,000 cases annually. Survival analysis indicates that without treatment or with limited access to it, mortality rates exceed 90% within 10–15 years after diagnosis (49). Because the present study aimed solely to shed light on the associated risk factors, it could not provide data regarding the mortality rate among infected cases.

Options for managing CE include surgery, percutaneous sterilization, medication, and observation. Early AE typically requires surgical treatment, but patients unsuitable for surgery or who have undergone surgical removal of parasite lesions must undergo prolonged treatment with benzimidazoles like albendazole or mebendazole (49, 53).

To effectively combat hydatid cyst disease, a comprehensive strategy is essential. This includes interrupting the transmission of Echinococcus from animals to people via deworming initiatives, appropriate disposal of animal remains, and maintaining good hygiene. Raising awareness through community education plays a significant role. Regular monitoring and cooperative actions are key to early detection. Timely intervention, including surgical procedures and medication, is crucial. Sustained control efforts depend on educating the populace and promoting collaboration. An integrated approach that combines prevention, monitoring, treatment, and education is vital for addressing hydatid cyst disease in developing countries (54).

## 5 Conclusion

Echinococcosis remains a significant public health issue, with higher infection rates linked to rural residency, lower education levels, agricultural work, dog ownership, inadequate hand hygiene, and alternative water sources. These findings highlight the need for targeted public health measures, such as educational initiatives and improved access to clean water and hygiene practices. Strengthened governmental guidelines addressing these risk factors could help reduce the global impact of echinococcosis.

## Data Availability

The original contributions presented in the study are included in the article/supplementary material, further inquiries can be directed to the corresponding author.
